# 艾曲泊帕治疗成人原发免疫性血小板减少症停药后疗效维持及预测因素

**DOI:** 10.3760/cma.j.issn.0253-2727.2023.01.006

**Published:** 2023-01

**Authors:** 慧平 孙, 建华 游, 秋生 陈, 瑾 王, 军民 李

**Affiliations:** 上海交通大学医学院附属瑞金医院血液科，上海血液学研究所，上海 200025 Department of Hematology, Institute of Hematology, Shanghai Jiao Tong University School of Medicine, Shanghai Rui Jin Hospital, Shanghai 200025, China

**Keywords:** 血小板减少, 艾曲泊帕, 治疗结果, Thrombocytopenia, Eltrombopag, Treatment outcome

## Abstract

**目的:**

观察艾曲泊帕对成人原发免疫性血小板减少症（ITP）的治疗反应和停药后疗效维持情况并探寻其预测因素。

**方法:**

纳入2013年6月14日至2021年5月31日期间在上海交通大学医学院附属瑞金医院血液科接受艾曲泊帕治疗的成人ITP患者，对其临床资料进行回顾性分析。随访截止时间为2021年12月31日。艾曲泊帕起始剂量为25 mg/d口服，最大剂量75 mg/d。按照血小板计数调整艾曲泊帕剂量，维持血小板计数（50～150）×10^9^/L。基于患者意愿和医生判断（处方用药）或者临床试验按照方案终止用药。对所有接受艾曲泊帕治疗的患者进行疗效评估，在治疗有效（疗效评估为完全反应或有效）并且减停艾曲泊帕的患者中进行无治疗有效（TFR）评估，对相关因素进行分析。

**结果:**

106例ITP患者纳入研究，男33例，女73例，中位年龄50（18～89）岁；新诊断ITP 2例，持续性ITP 10例，慢性ITP 94例。艾曲泊帕治疗后完全反应率为44.3％（47/106），有效率为34.0％（36/106），总反应率（ORR）为78.3％（83/106）。83例治疗有效患者经过艾曲泊帕停药，81例可评估停药后疗效，17例（21.0％）获得TFR。获得TFR的患者中位随访时间126（30～170）周，复发率为17.6％（3/17），无复发生存率（RFS）为76.5％。单因素分析结果显示，停止其他ITP合并治疗后无复发（*P*＝0.001）、艾曲泊帕开始减停时血小板计数≥100×10^9^/L（*P*＝0.007）、艾曲波帕开始减停时剂量≤25 mg/d（*P*＝0.031）与有效持续时间延长相关。多因素分析结果显示，停止其他ITP合并治疗后无复发与有效持续时间延长相关（*P*＝0.002）。

**结论:**

艾曲泊帕对成人ITP患者有效且部分患者可获得TFR。停止其他ITP合并治疗后无复发、艾曲泊帕开始减停时PLT≥100×10^9^/L以及艾曲泊帕开始减停时剂量≤25 mg/d是获得TFR的预测因素。

## Abstract (en)

**Objective:**

To determine the efficacy of eltrombopag for primary immune thrombocytopenia（ITP）in adults and the predictive factors for treatment-free response（TFR）.

**Methods:**

Clinical data of adults with ITP who received eltrombopag from June 14, 2013 to May 31, 2021 in the Hematology Department of Ruijin Hospital affiliated with Shanghai Jiao Tong University Medical College were retrospectively analyzed. The initial dose of eltrombopag was 25 mg/d, and the maximum dose was 75 mg/d; the dose was adjusted to maintain the platelet count to within 50-150×10^9^/L. Treatment was discontinued according either to the protocol, on the patient's wishes or doctor's judgment（prescription medication）, or based on clinical trials. The efficacy of eltrombopag and factors for TFR among patients who achieved complete response and those who discontinued treatment were analyzed.

**Results:**

Overall, 106 patients with ITP（33 men and 73 women）were included in the study. The median age of patients was 50（18–89）years. There were 2, 10, and 94 cases of newly diagnosed, persistent, and chronic ITP, respectively. The complete response rate was 44.3％（47/106）, the response rate was 34.0％（36/106）, and the overall response rate was 78.3％（83/106）. Meanwhile, 83 patients who responded to treatment discontinued eltrombopag; of these, 81 patients were evaluated. Additionally, 17 patients（21.0％）achieved TFR. The median follow-up duration of patients who achieved TFR was 126（30–170）weeks. The recurrence rate was 17.6％（3/17）, and the relapse-free survival rate was 76.5％. The results of univariate analysis revealed that non-recurrence after discontinuation of other treatments for ITP（*P*＝0.001）, and platelet count and eltrombopag dose of ≥100×10^9^/L（*P*＝0.007）and ≤25 mg/d（*P*＝0.031）, respectively, upon discontinuation of eltrombopag were predictors of TFR; these effects were attributed to prolonged effective duration of eltrombopag. Multivariate analysis showed that there was a correlation between non-recurrence and prolonged effective duration after discontinuation of other treatments for ITP（*P*＝0.002）.

**Conclusion:**

Eltrombopag is effective for patients with ITP as it can result in TFR. Predictors for TFR include non-recurrence after discontinuation of concomitant ITP treatment, and platelet count and eltrombopag dose of ≥100 × 10^9^/L and ≤25 mg/d upon discontinuation of treatment, respectively.

原发性免疫性血小板减少症（ITP）是由免疫调节失衡引起的自身免疫性疾病。ITP患者异常的体液和细胞免疫除了导致血小板过度破坏外，还抑制巨核细胞的增殖分化，从而使血小板生成减少[1]。既往应用的糖皮质激素、静脉注射免疫球蛋白、免疫抑制剂和脾切除等都是通过减少血小板过度破坏而达到治疗目的，但疗效有限或不良反应大而不适合长期应用[2]。艾曲波帕是一种新型的血小板生成素受体激动剂（TPO-RA），二线治疗ITP患者的6个月有效率达79％[3]，疗效持久且安全[4]。中国临床试验（TRA113765）结果显示，对于糖皮质激素或其他治疗无效的ITP患者，艾曲波帕的总有效率（ORR）达57.7％[5]。但大部分患者在艾曲泊帕治疗有效后停药2周内出现疾病复发[3]。因此，近年来的临床研究关注TPO-RA在治疗取得疗效后停药的可能性和相关预测因素[6]–[8]。本中心艾曲波帕中国临床试验（TRA113765）结果显示，艾曲泊帕停药后疗效维持的患者比例为21.4％[9]。本研究对在本中心ITP患者艾曲泊帕治疗后减停药结果进行回顾性分析，旨在明确艾曲泊帕治疗成人ITP的治疗反应和停药后疗效维持情况并探寻相关影响因素。

## 病例与方法

一、研究对象

本研究为单中心回顾性研究，数据来源于艾曲泊帕中国临床试验（TRA113765）和中国上市后处方用药的患者资料。病例收集起止日期为2013年6月14日至2021年5月31日，随访截止时间为2021年12月31日。排除①无艾曲波帕停药；②在艾曲波帕或合并ITP治疗药物减停过程中因疾病复发而增加艾曲波帕剂量的患者。患者诊断、分期及疗效判断均参照《成人原发免疫性血小板减少症诊断与治疗中国指南（2020年版）》[10]：①新诊断ITP：确诊后3个月以内的患者；②持续性ITP：确诊后3～12个月血小板持续减少的患者，包括未自发缓解和停止治疗后不能维持完全反应（CR）的患者；③慢性ITP：血小板持续减少超过12个月的患者。疗效判断标准：①CR：治疗后血小板计数≥100×10^9^/L且无出血表现。②有效（R）：治疗后血小板计数≥30×10^9^/L，比基础血小板计数增加至少2倍且无出血表现。③无效（NR）：治疗后血小板计数<30×10^9^/L，或血小板计数增加不到基础值的2倍，或有出血。④复发：治疗有效后血小板计数降至30×10^9^/L以下，或降至不到基础值的2倍，或出现出血症状。在定义CR、R或复发时，至少检测2次血小板计数，间隔至少7 d。

停药后疗效相关指标定义：①无治疗有效（treatment-free response, TFR）：停用艾曲泊帕及其他ITP治疗药物后血小板计数≥50×10^9^/L持续≥6个月。②有效持续时间：自停止艾曲泊帕及其他ITP治疗药物，血小板计数≥50×10^9^/L起始日至疾病复发或随访截止的时间。艾曲波帕减药过程中疾病复发者，有效持续时间为0。

二、研究方法

艾曲泊帕用药方法按照临床试验方案或药物说明书，起始剂量为25 mg/d口服，最大剂量≤ 75 mg/d。按照血小板计数调整艾曲泊帕剂量，使血小板计数维持（50～150）×10^9^/L。基于患者意愿和医生判断（处方用药）停药，或按照临床试验方案终止用药。

对治疗有效（CR或R）且伴其他ITP合并治疗的患者，减停药物分两个阶段：①第一阶段：减停合并治疗药物，以维持血小板计数波动≤15％并无复发为目标；若血小板计数波动>15％或疾病复发，则停止减量或用药恢复至复发前剂量，待血小板计数稳定后进入第二阶段；②第二阶段：完成第一阶段调整合并用药后，每2周调整1次艾曲泊帕剂量，按75 mg/d→50 mg/d→25 mg/d→减少用药频次的模式减量，以维持血小板计数波动≤15％并无复发；若PLT计数波动>15％但无复发，则调整减药剂量，缓慢减药。无其他ITP合并治疗的患者直接进入第二阶段。部分患者在艾曲波帕治疗停止时无逐渐减量的过程，直接终止治疗，这些患者包括所有临床试验受试者和部分处方用药按意愿停药的患者。

对所有接受艾曲泊帕治疗患者进行疗效评估，对治疗有效（CR或R）并且停药患者进行随访，评估TFR率，并按TFR状况将患者分为两组，对患者的基线状况和治疗相关因素进行比较并分析影响有效持续时间的相关因素。

三、观察指标

主要研究指标为TFR率。次要研究指标：①获得TFR者基线特征和治疗相关指标；②有效持续时间影响因素；③无复发生存（RFS）：对获得TFR患者，观察从停药开始直至ITP复发或随访截止日所持续时间。

四、统计学处理

按意向治疗（intent-to-treat, ITT）统计。所有接受艾曲泊帕治疗并减停药的患者均纳入分析。计量资料以“中位数（范围）”或“平均数”表示，计数资料以“比例”或“比率”表示。两组分类或反应率比较单因素分析采用非参数检验（*χ*^2^检验或Fisher精确概率法），计量资料采用两样本*t*检验。应用Cox比例风险模型分析相关因素对有效持续时间的影响，Kaplan-Meier生存分析无复发生存率。*P*<0.05为差异有统计学意义。统计应用SPSS26.0软件，生存曲线及森林图绘制应用GraphPad Prism9.0。

## 结果

一、一般资料

共有106例患者纳入本研究，其中艾曲波帕中国临床试验（TRA113765）入组患者15例，中国上市后处方用药91例。男33例，女73例，中位年龄50（18～89）岁。疾病分期：新诊断2例，持续性10例，慢性94例。根据是否获得TFR分为2组，组间基线临床指标比较差异无统计学意义，详见表1。

**表1:** 艾曲泊帕停药后获得、未获得无治疗有效（TFR）原发免疫性血小板减少症患者的基线特征比较

指标	TFR组（17例）	非TFR组（64例）	统计量	*P*值
中位年龄［岁，*M*（范围）］	35（19～82）	52（18～89）	1.216	0.228
年龄≥65岁［例（％）］	3（17.6）	11（17.2）	0.002	0.965
性别［例（％）］			0.064	0.800
男	4（23.5）	17（26.6）		
女	13（76.5）	47（73.4）		
疾病分期［例（％）］			0.303	0.582
新诊断+持续性	3（17.6）	8（12.5）		
慢性	14（82.4）	56（87.5）		
既往治疗［例（％）］			1.074	0.584
1线	9（52.9）	33（51.6）		
2线	5（29.4）	13（20.3）		
≥3线	3（17.6）	18（28.1）		
脾切除	0（0）	2（3.1）		
基线PLT［×10^9^/L，*M*（范围）］	22（8～28）	20（7～48）	0.192	0.848
出血评分［*M*（范围）］	1（0～6）	1（0～6）	-0.509	0.612
确诊至艾曲泊帕治疗时间［月，*M*（范围）］	20（2～50）	30（1～130）	-1.919	0.059
合并其他治疗［例（％）］	13（76.5）	54（84.3）	0.587	0.444

二、疗效

全部106例患者中，治疗后达到CR、R的患者分别为47例（44.3％）、36例（34.0％），ORR为78.3％（83/106），23例（21.7％）疗效评估为NR。

三、TFR

83例治疗有效（获得CR或R）患者停止艾曲泊帕治疗，其中2例因停药后即转换治疗方案而被剔除（1例行脾切除术，1例临床试验患者更新诊断为继发免疫性血小板减少症并调整为免疫抑制剂治疗），故可评估有效持续时间的患者81例，其中14例（临床试验TRA113765）按临床试验方案停药（终止），67例为中国上市后处方用药。处方用药患者的停药原因：患者意愿50例（74.6％），因病情稳定医生建议10例（14.9％），围手术期用药6例（9.0％），不良反应（转氨酶升高Ⅲ级）1例（1.5％）。随访结果显示，17例（21.0％）患者获得TFR。获得TFR患者的中位随访时间为126（30～170）周，其中3例（17.6％）分别于停药后30、74、130周复发，Kaplan-Meier生存分析显示RFS率为76.5％。无复发生存曲线见图1。按是否获得TFR将患者分为两组，下列指标在两组间存在显著差异：①停止其他ITP合并治疗后无复发患者比例：TFR组、非TFR组分别为100％、48.4％（*P*＝0.001）；②艾曲波帕开始减停时血小板计数≥100×10^9^/L患者比例：TFR组、非TFR组分别为94.1％、45.3％（*P*<0.001）；③艾曲波帕开始减停时中位血小板计数：TFR组、非TFR组分别为126（65～185）×10^9^/L、82（32～165）×10^9^/L（*P*<0.001）。详见表2。

**图1:**
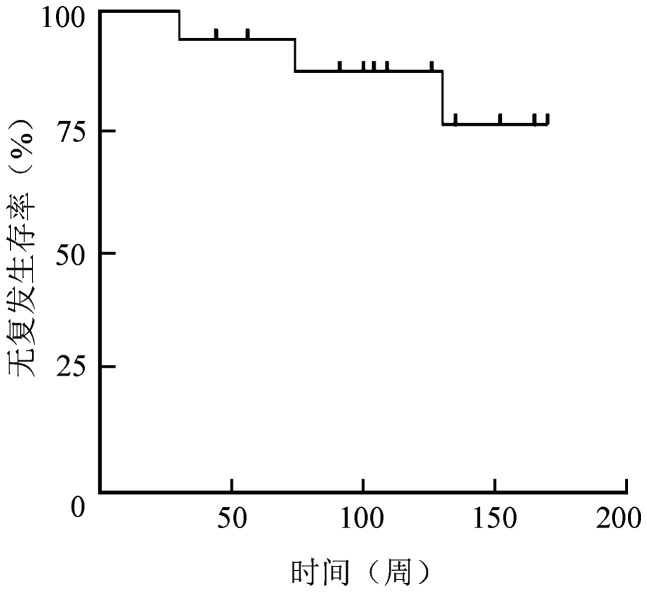
17例艾曲泊帕停药后获得无治疗有效（TFR）原发免疫性血小板减少症患者的无复发生存曲线

**表2:** 艾曲泊帕停药后获得、未获得无治疗有效（TFR）原发免疫性血小板减少症患者的治疗相关指标

指标	TFR组（17例）	非TFR组（64例）	统计量	*P*值
艾曲泊帕治疗第14天PLT≥50×10^9^/L［例（％）］	15（88.2）	46（71.9）	1.933	0.164
艾曲泊帕减停药前PLT≥100×10^9^/L［例（％）］	16（94.1）	29（45.3）	12.958	<0.001
艾曲泊帕减停药前PLT［×10^9^/L，*M*（范围）］	126（65～185）	82（32～165）	4.443	<0.001
艾曲泊帕停药方式［例（％）］			0.321	0.984
终止	3（16.7）	19（30.2）		
减停	14（83.3）	45（69.8）		
艾曲泊帕减停药平均剂量［mg/d，*x±s*］	29.4±13.2	35.5±16.6	−1.411	0.162
艾曲泊帕减停前剂量≤25 mg/d［例（％）］	15（88.2）	43（67.2）	2.927	0.087
艾曲泊帕疗程［月，*M*（范围）］	6（3～65.7）	6（0.5～65.9）	0.897	0.372
艾曲泊帕疗程≥6个月［例（％）］	14（82.4）	49（76.5）	0.261	0.610
艾曲泊帕减停药持续时间［月，*M*（范围）］	3（0～10）	2.5（0～12）	0.737	0.463
其他合并治疗停药率［％（停药例数/合并治疗例数）］	100（13/13）	38.9（21/54）	15.655	<0.001

四、有效持续时间的影响因素

单因素分析包括如下指标：性别（男/女）、年龄（是否≥65岁）、基线血小板计数（是否≤15×10^9^/L）、疾病分期（是否慢性）、治疗线数（是否≥2线）、艾曲泊帕治疗第14天血小板计数（是否≥50×10^9^/L）、艾曲泊帕疗程（是否≥6个月）、艾曲泊帕减量持续时间（是否≤3个月）、合并其他ITP治疗（有无）、艾曲泊帕减停开始血小板计数（是否≥100×10^9^/L）、停药方式（终止或减停）、艾曲泊帕减停开始时剂量（是否≤25 mg/d）、停止其他ITP合并后疾病状态（是否复发）。单因素分析结果显示，停止其他ITP合并治疗后无复发（*P*＝0.001）、艾曲泊帕开始减停时血小板计数≥100×10^9^/L（*P*＝0.007）、艾曲泊帕开始减停时剂量≤25 mg/d（*P*＝0.031）与有效持续时间延长相关。多因素分析结果显示停止其他ITP合并治疗后无复发与有效持续时间延长相关（*P*＝0.002），详见图2。

**图2:**
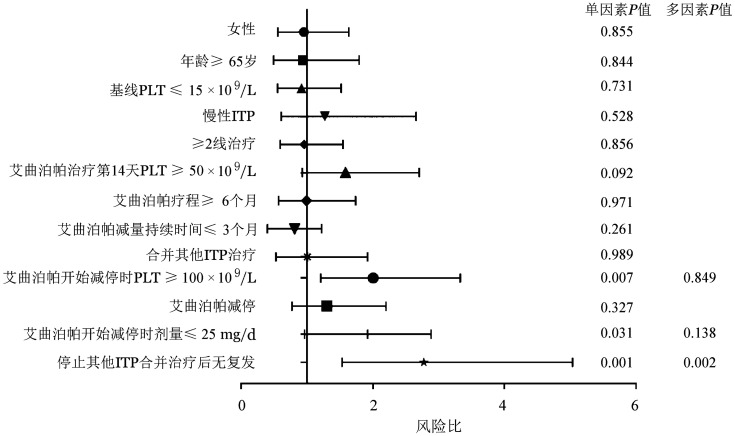
原发免疫性血小板减少症（ITP）患者艾曲泊帕停药后有效持续时间相关因素分析森林图

## 讨论

本研究88.7％（94/106）的患者为慢性ITP，而慢性ITP的病情并不能随时间延长获得进一步的自发缓解[11]。本回顾性研究结果显示，艾曲泊帕治疗经治ITP患者的ORR达到78.3％，其中CR率为44.3％，与中国临床试验（TRA13765）[5],[9]结果近似。本中心患者治疗的药物剂量调整方法参照临床试验（TRA13765）方案，即药物调整过程中维持血小板计数波动≤15％，减停药物顺序为先减停其他ITP合并治疗，再减停艾曲波帕。目前临床研究关注患者在获得临床疗效后如何进行药物调整和停药的可能性[6]–[8]，本研究参照这些研究报告对相关指标的定义，将TFR定义为停止艾曲泊帕及其他ITP合并治疗后血小板计数≥50×10^9^/L并持续≥6个月。本研究结果显示TFR率为21％，与Lucchini等[7]报告的结果相似，也与本中心中国临床试验（TRA113765）[9]报告的停药后疗效维持率一致，对获得TFR者的随访结果显示，在中位随访期126周内，疾病复发率为17.6％（3/17），RFS为76.5％，提示获得TFR患者能够维持长期有效，复发率较低。

本项研究中，处方用药的患者中74.6％的患者减停药物是按照患者意愿，停药的主要原因是考虑治疗相关费用以及长期用药的不良反应。在减停其他合并治疗时复发的患者，部分选择停艾曲波帕并转换治疗方案，有效持续时间判断为0，另有部分选择增加艾曲波帕剂量，收集病例时剔除。这些按意愿减停药物的患者中77.8％（63/81）用药时间≥6个月。尽管不同研究中艾曲泊帕的疗程存在差异，但停药后的有效维持率基本一致。本组病例中，艾曲波帕疗程对TFR无显著影响。两组比较发现，TFR组血小板计数水平中位数高于非TFR组（126×10^9^/L对82×10^9^/L，*P*<0.001）、CR患者比例显著增高（94.1％对45.3％，*P*<0.001）以及合并ITP治疗停药率高（100％对38.9％，*P*＝0.001）。这些指标均与药物治疗的疗效相关，提示治疗反应对TFR有影响。

研究发现，艾曲泊帕除了促进巨核细胞增殖分化和血小板生成以外，还对免疫功能具有调节作用。艾曲泊帕的作用机制包括下列几个方面：①艾曲泊帕促进巨核细胞增殖分化和前血小板生成：ITP患者与正常人相比，产板巨核细胞显著减少，特别是慢性和持续性ITP患者尤为显著[12]。艾曲泊帕作为TPO受体激动剂，与TPO受体跨膜区结合后通过激活下游STAT3/5、AKT和ERK信号通路，促进巨核细胞增殖分化和前血小板生成[13]，从而使血小板计数升高，达到疾病临床缓解。②提高免疫耐受性：免疫耐受缺失导致免疫失平衡是ITP发病的重要机制，在这个复杂的过程中，调节性T细胞（Treg）被认为是有重要作用的。艾曲泊帕治疗有效的患者，Treg功能得到恢复，TGF-β1水平升高[14]–[15]。另外，ITP发病与免疫球蛋白Fcγ受体（FcγR）异常有关。FcγRⅡb能激发对免疫细胞抑制作用，研究者发现TPO-RA通过刺激单核细胞FcγRⅡb表达，从而消除免疫抑制和活化状态不平衡，发挥免疫抑制作用和拮抗致病性的免疫异常活化，抑制抗血小板抗体产生[16]–[17]。艾曲波帕通过调节免疫耐受和刺激巨核细胞增殖分化产生血小板而使疾病获得缓解。

本研究发现，停止其他ITP合并治疗后无复发、艾曲波帕开始减停时PLT≥100×10^9^/L、艾曲波帕开始减停时剂量≤25 mg/d是获得TFR的预测因素（*P*值分别为0.002、0.007、0.031），多因素分析进一步确定停止合并ITP治疗无复发是获得长期持续有效的相关因素。这些相关因素均与艾曲波帕疗效相关，获得良好疗效的患者有效治疗剂量较低（≤25 mg/d）、获得CR（血小板计数≥100×10^9^/L）并保持稳定，在艾曲波帕治疗有效后，免疫功能和血小板生成功能得到恢复，可以停止其他ITP合并治疗药物。因此，艾曲波帕疗效相关指标与TFR和长期持续有效相关，是获得TFR的预测因素。

综上所述，本研究结果显示，艾曲泊帕对ITP患者有一定疗效（ORR达78.3％）且部分患者可获得TFR，获得TFR患者的RFS达76.5％。停止其他ITP合并治疗后无复发、艾曲泊帕减停开始时PLT≥100×10^9^/L以及艾曲波帕剂量≤25 mg/d是获得TFR的预后因素。本研究结果可为合理设计前瞻性临床研究方案提供依据。本研究为单中心回顾性观察研究，患者例数较少且以慢性ITP为主，未能反映疾病早期应用艾曲波帕对长期疗效的影响，需要通过扩大样本量以进一步分层分析。
